# Absence of Both IL-7 and IL-15 Severely Impairs the Development of CD8^+^ T Cell Response against *Toxoplasma gondii*

**DOI:** 10.1371/journal.pone.0010842

**Published:** 2010-05-26

**Authors:** Rajarshi Bhadra, Hongbing Guan, Imtiaz A. Khan

**Affiliations:** 1 Department of Microbiology, Immunology and Tropical Medicine, George Washington University, Washington, D. C., United States of America; 2 Department of Pathology, Microbiology and Immunology, University of South Carolina School of Medicine, Columbia, South Carolina, United States of America; Institut Jacques Monod, France

## Abstract

CD8^+^ T cells play an essential role in the protection against both acute as well as chronic *Toxoplasma gondii* infection. Although the role of IL-15 has been reported to be important for the development of long-term CD8^+^ T cell immunity against the pathogen, the simultaneous roles played by both IL-15 and related γ-chain family cytokine IL-7 in the generation of this response during acute phase of infection has not been described. We demonstrate that while lack of IL-7 or IL-15 alone has minimal impact on splenic CD8^+^ T cell maturation or effector function development during acute Toxoplasmosis, absence of both IL-7 and IL-15 only in the context of infection severely down-regulates the development of a potent CD8^+^ T cell response. This impairment is characterized by reduction in CD44 expression, IFN-γ production, proliferation and cytotoxicity. However, attenuated maturation and decreased effector functions in these mice are essentially downstream consequences of reduced number of antigen-specific CD8^+^ T cells. Interestingly, the absence of both cytokines did not impair initial CD8^+^ T cell generation but affected their survival and differentiation into memory phenotype IL-7Rα^hi^ cells. Significantly lack of both cytokines severely affected expression of Bcl-2, an anti-apoptotic protein, but minimally affected proliferation. The overarching role played by these cytokines in eliciting a potent CD8^+^ T cell immunity against *T. gondii* infection is further evidenced by poor survival and high parasite burden in anti IL-7 treated IL-15^−/−^ mice. These studies demonstrate that the two cytokines, IL-7 and IL-15, are exclusively important for the development of protective CD8^+^ T cell immune response against *T. gondii*. To the best of our knowledge this synergism between IL-7 and IL-15 in generating an optimal CD8^+^ T cell immunity against intracellular parasite or any other infectious disease model has not been previously reported.

## Introduction

Development of effective CD8^+^ T cell response is critical for the control of various intracellular pathogens including, *T. gondii*, an obligate intracellular parasite [1], [2]. Although both CD4^+^ and CD8^+^ T cell response is induced during acute *T. gondii* infection, long-term protection against the parasite is primarily dependent on CD8^+^ T cell subset [3]. Immune CD8^+^ T cells from *T. gondii* infected host are important source of IFN-γ, a cytokine which is critical for survival against both acute as well as chronic phases of infection [3]. Moreover, CD8^+^ T cells from *T. gondii* infected hosts have the ability to exhibit in vitro cytotoxic activity against parasite-infected targets [4]. The cytotoxic function of these antigen-specific CD8^+^ T cells has been reported to play an important role in keeping chronic infection under control [5]. Depletion of either IFN-γ or CD8^+^ T cells abrogates protective immunity against *T. gondii* infection leading to morbidity or mortality of infected host.

Although importance of CD8^+^ T cell immunity against *T. gondii* infection is well established, the cytokines precisely involved in generating this response have not been well demonstrated. Previous studies from our laboratory have reported that exogenous treatment of infected mice with IL-7 augments CD8^+^ T cell response against *T. gondii* resulting in their ability to survive lethal infection [6]. In subsequent studies we reported that neutralization of endogenous IL-15 compromises memory CD8^+^ T cell response in the *T. gondii* infected animals which lose their ability to survive re-challenge [6], [7]. IL-7, IL-15 and IL-2 are members of the γ-chain family of cytokines that have been implicated in the process of memory CD8^+^ T cell generation [2], [8], [9]. While IL-7 plays an important role in providing survival signals to naïve and memory CD8^+^ T cells, IL-15 is believed to be crucial for driving basal proliferation of memory CD8^+^ T cells [10]. Although the role of IL-15 in memory CD8^+^ T cell development during Toxoplasma infection has been addressed, its importance in the induction of CD8^+^ T cell response during acute phase has not been evaluated [7], [11], [12]. Similarly while a previous study from our laboratory using exogenous IL-7 treatment showed increased CTL response in *T. gondii* infected mice, importance of endogenous cytokine in eliciting this response has not been well studied [6]. Moreover, the roles of both IL-7 and IL-15 in the induction of primary CD8^+^ T cell response has not been evaluated in models of primary infection. A study by Chang *et al* which demonstrates that effector and memory T cell lineage imprinting can occur as early as the first cell division underscores the need for understanding the determinants of CD8^+^ T response during the early phase of the response [13]. In the present study we assayed the development of primary CD8^+^ T cell immunity against *T. gondii* infection in IL-15 deficient mice that are depleted of IL-7. We demonstrate that while lack of IL-7 or IL-15 alone had minimal effect on CD8^+^ T cell immunity, the absence of both cytokines severely impacted this response against the pathogen.

## Materials and Methods

### Mice, infections and antibody treatment

**Figure 1:**
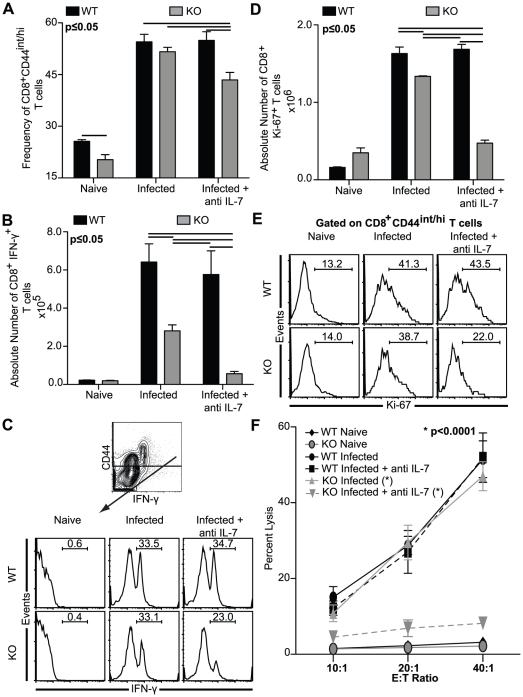
Impaired CD8^+^ T cell response in anti IL-7 treated KO mice. Splenocytes were harvested from WT, KO, anti IL-7 treated WT and anti IL-7 treated KO mice at day 14 p.i. *A*, Percentage of CD44^int/hi^ cells in the CD8^+^ T cell population depicted as bar graph was enumerated by surface staining the splenocytes for CD8β and CD44. *B–C*, For IFNγ production, cells were stimulated in vitro with TLA and after overnight incubation stained for CD8β, CD44 and IFN-γ as described in *Materials and Methods*. Data are presented as absolute number of IFN-γ producing CD8^+^ T cells (*B*) or as frequency of CD8^+^CD44^int/hi^ cells expressing IFN-γ (*C*). *C*, The CD44 vs. IFN-γ plot in the *top panel* is gated on CD8^+^ T cells. *D–E,* For evaluation of cycling CD8^+^ T cells, splenocytes were stained for CD8β, CD44, and Ki-67. Data are presented as absolute number of CD8^+^Ki-67^+^ T cells (*D*) or as histograms gated on CD8^+^CD44^int/hi^ T cells (*E*). *F*, CTL assay was performed with purified splenic CD8^+^ T cells cultured in presence of TLA and irradiated feeder cells. Following incubation with ^51^Cr labeled infected targets at various E:T ratios, radioisotope release was assayed. The data depicted here represents at least 2 experiments with 3–4 mice per group.

Animal studies were carried out in agreement with Institutional Animal Care and Use Committee approved guidelines at George Washington University Medical Center. 6 to 8 week old female IL-15^−/−^ mice (Taconic Farms) and C57BL/6 mice (NCI) were infected per-orally with 10 cysts of ME49 strain (Figure. 1–6) of *T. gondii*. CD8^+^ T cell responses were evaluated at day 14 post-infection (p.i.). Anti IL-7 antibody (M25) was a kind gift from Amgen. The antibody was injected into wild-type or IL-15^−/−^ i.p. (intraperitoneally) at 0.5 mg per mouse at 3-day intervals. The treatment was initiated one day before infection and continued till termination of experiment. Control mice were injected with equal volume of saline. For some experiments, IL-15^−/−^ and anti IL-7 treated IL-15^−/−^ animals were infected i.p. with 1×10^6^ cps1-1 parasites (Figure. 7) and CD8^+^ T cell responses were evaluated at day 14 p.i..

**Figure 2 pone-0010842-g002:**
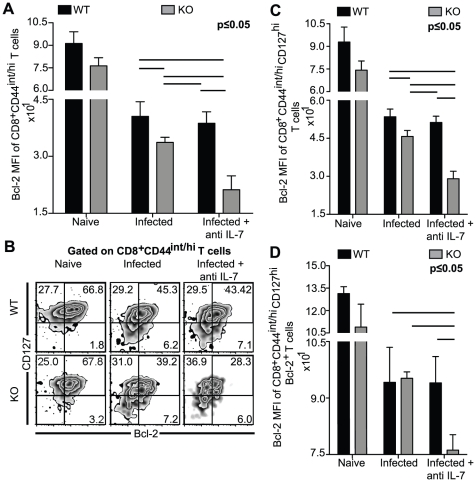
Simultaneous deficiency of IL-7 and IL-15 results in poor development of IL-7Rα^hi^Bcl-2^+^CD8^+^ T cells. WT, KO, anti IL-7 treated WT and anti IL-7 treated KO mice were sacrificed at day 14 p.i. *A–D*, Splenocytes were surface stained for CD8β, CD44 and CD127, followed by intracellular staining for Bcl-2. *A*, Bcl-2 MFI of CD8^+^CD44^int/hi^ T cells is shown as bar graph. *B–D*, Data of activated CD8^+^ T cell subsets are presented as Bcl-2 vs. CD127 density-contour plot (*B*) or as bar graphs of Bcl-2 MFI of CD8^+^CD44^int/hi^CD127^hi^ T cells (*C*) or CD8^+^CD44^int/hi^CD127^hi^Bcl-2^+^ (*D*). The experiment was performed at least twice with similar results. The data is representative of one experiment with 4 mice per group.

**Figure 3 pone-0010842-g003:**
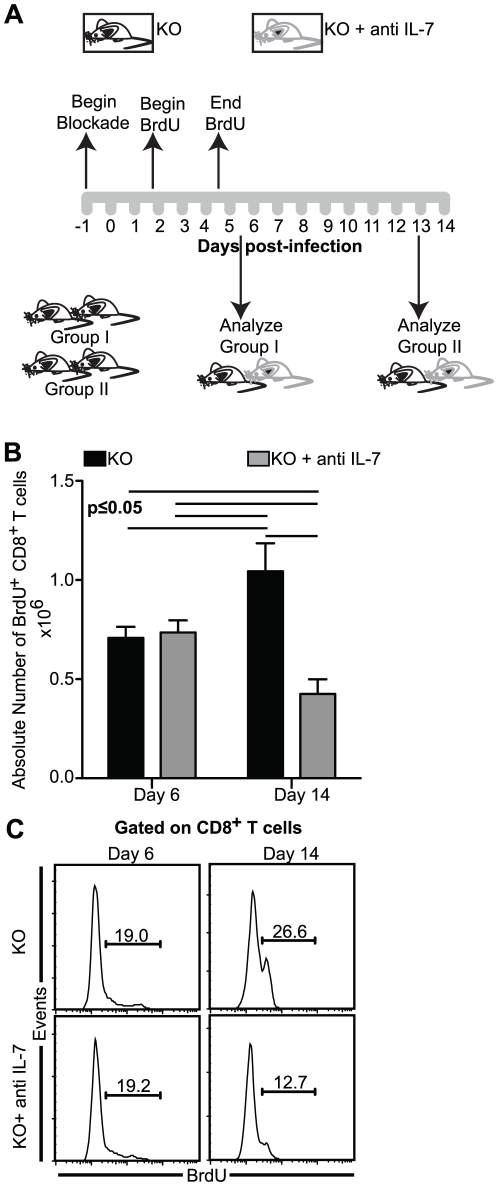
Lack of IL-7 and IL-15 results in suboptimal expansion of BrdU labeled CD8^+^ T cells. *A*, KO mice treated with anti IL-7 or saline were injected with BrdU i.p. daily between day 2 and day 5 p.i. Splenocytes were harvested at day 6 and day 14 p.i. and analyzed for BrdU labeled CD8^+^ T cells as stated in *Materials and Methods*. *b–c*, Data are presented as absolute number (*B*) or percentage (*C*) of BrdU labeled of CD8^+^ T cells. The data depicted represents one of two experiments with similar results. Each group in the experiment had at least 3 mice.

**Figure 4 pone-0010842-g004:**
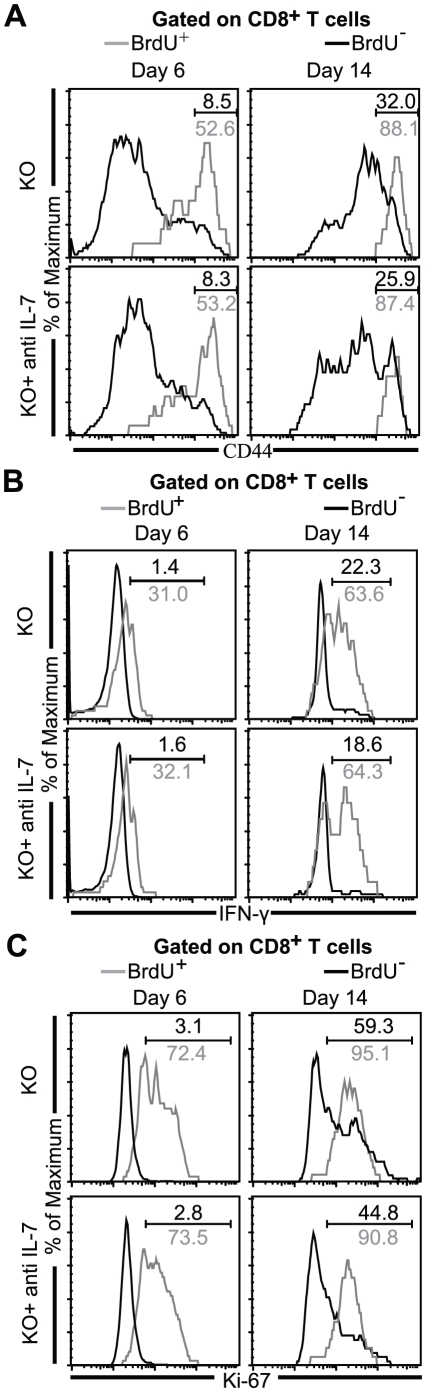
BrdU labeled CD8^+^ T cells in IL-7 depleted KO mice show unimpaired response. Splenocytes from BrdU injected anti IL-7 or saline treated mice KO were harvested at day 6 and day 14 p.i. Surface and intracellular staining was performed as described in *Materials and Methods*. *a–c*, Data are presented as histograms depicting the percentage of CD44^hi^ (*A*), IFN-γ (*B*) and Ki-67 (*C*) expression in BrdU^+^ and BrdU^−^ CD8^+^ T cells. *A–C*, The data shown here is representative of one of two similar experiments with 3–4 mice per group.

**Figure 5 pone-0010842-g005:**
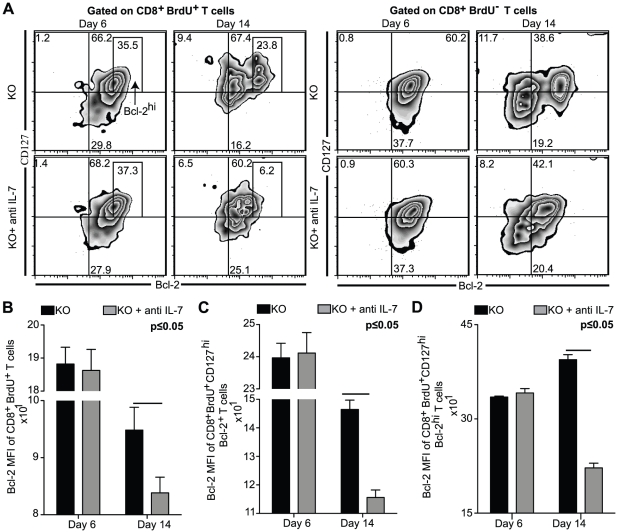
Bcl-2 and IL-7Rα are downregulated in BrdU^+^CD8^+^ T cells from IL-7 depleted KO mice. Splenocytes from BrdU injected antibody or saline treated KO mice were harvested at day 6 and day 14 p.i. The cells were stained for CD8β, CD127, Bcl-2 and BrdU as described in *Materials and Methods*. *A*, The Bcl-2 vs. CD127 density-contour plots depicted on the *top panel* are gated on CD8^+^Brdu^+^ T cells (*top left panel*) and CD8^+^BrdU^−^ T cells (*top right panel*). *B–D*, Bcl-2 MFI of CD8^+^BrdU^+^ T cells (*B*), CD8^+^BrdU^+^CD127^hi^Bcl2^+^ T cells (*C*) and CD8^+^BrdU^+^CD127^hi^Bcl2^hi^ T cells (*D*) are presented as bar graphs in the *lower panel*. The experiment was performed twice with similar results. The data is representative of one of two similar experiments with 3–4 mice per group.

**Figure 6:**
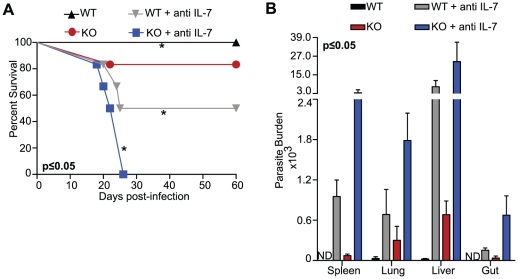
IL-7 depleted KO mice rapidly succumb to oral Toxoplasma infection. WT and KO mice treated with anti IL-7 antibody or saline were infected per-orally with 10 cysts of *T. gondii. A*, Survival was monitored on a daily basis and statistical significance was calculated by Mantel-Cox Test. *B*, Parasite burden was measured in liver, lung, spleen and gut by quantitative PCR at day14 post-infection. Differential parasite burden in each tissue is statistically significant. The experiment was repeated twice with similar results and the data depicted is representative of one of two experiments with 4–6 mice per group.

**Figure 7:**
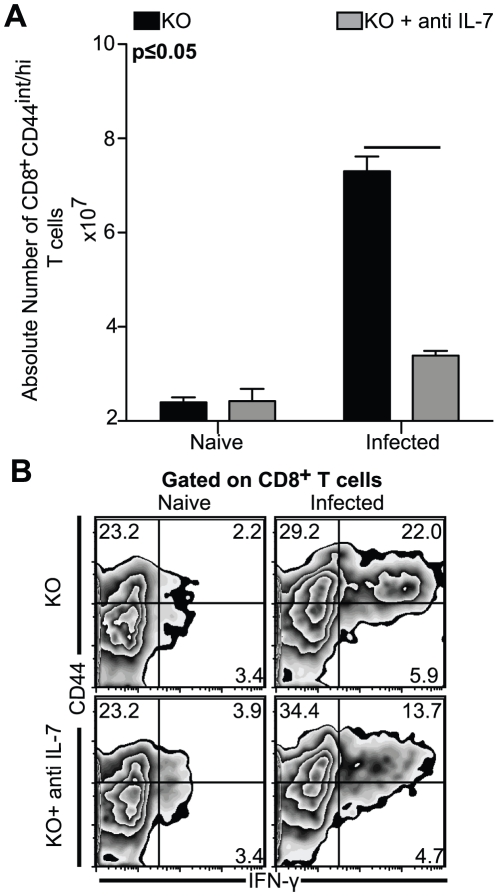
Cps vaccination *elicits a sub-optimal CD8^+^ T cells response in IL-7 depleted KO mice.* Splenocytes were harvested from KO and anti IL-7 treated KO mice infected with 1×10^6^ parasites of cps1-1 at day 14 p.i. *A*, Absolute number of CD8^+^CD44^int/hi^ T cells present in untreated or antibody treated infected or naïve KO mice is shown in the top panel. *B*, For evaluation of cytokine response splenocytes were stimulated in anti CD3 coated plates and then stained for CD8β, CD44 and IFN-γ as described in *Materials and Methods*. The experiment was repeated twice with similar results and the data depicted is representative of one of two experiments with 3–4 mice per group.

### Toxoplasma Lysate Antigen (TLA) preparation

TLA was extracted from RH strain of parasite and preparation was made as previously described [14].

### Lymphocyte isolation, cell surface staining and intracellular staining

Lymphocytes were isolated from spleen or liver as reported earlier [11]. Antibodies for detection of IL-7Rα (CD127; A7R34), CD8β (H35-17.2), CD44 (IM7), IL-2 (JES6-5H4), Eomesodermin (Eomes; 21Mags8), NK1.1 (PK136) and IFN-γ (XMG1.2) were purchased from eBioscience. Antibodies against CD3 (145-2C11), Ki-67 (B56), Bcl-2 (3F11), IL-12p40 (C15.6), IL-12Rβ1 (114) and IL-12Rβ2 (HAM10B9) were purchased from BD Biosciences. Antibody for T-bet (4B10) detection was purchased from Santa Cruz Biotechnology. For cytokine detection, restimulation was carried out for 12 h with 30 µg/ml of Toxoplasma lysate antigen (TLA) in supplemented DMEM at 37° C in 5% CO_2_. 1 µl/ml of monensin (BD Biosciences) was added during the final 8 h of stimulation. This was followed by surface staining and then intracellular staining as described before [7]. IL-12 detection was similarly performed but without exogenous TLA stimulation. For IFNγ detection by anti CD3 stimulation, splenocytes were plated on anti CD3 (BD Biosciences) coated plates for 4 h with 0.65 µl/ml brefeldin A (BD Biosciences) and 0.65 µl/ml monensin (BD Biosciences) and staining was performed as above. Cell fluorescence was measured with a FACSCalibur (BD Biosciences) cytometer and data was analyzed using FlowJo (TreeStar, Inc.) software. Numbers in histograms or dot plots indicate percentage unless otherwise mentioned. MFI denotes Mean Fluorescence Intensity.

### BrdU injection and staining

BrdU (BD Biosciences) was injected at 1 mg/mouse i.p. once daily between day 2 and day 5 p.i.. Splenocytes were analyzed for BrdU incorporation at day 6 and day 14 p.i.. Surface staining was followed by intracellular staining. The latter was performed using FITC BrdU Flow Kit (BD Biosciences) as per manufacturer's protocol. For cytokine detection restimulation was performed using a modified protocol. Restimulation was carried out for 15 h with 30 µg/ml of Toxoplasma lysate antigen (TLA) in supplemented DMEM at 37° C in 5% CO_2_. 0.65 µl/ml of brefeldin A (BD Biosciences) and 0.65 µl/ml monensin (BD Biosciences) was added during the final 9 h of stimulation.

### Quantification of Parasite Load

Quantification of parasite load in spleen, lung, gut and liver was performed at day 14 p.i. as described earlier [11]. DNA was isolated from tissues with the Qiamp tissue kit (QIAGEN, Valencia, CA) according to the manufacturer's instructions. Parasite DNA was amplified with primers specific for a 35-fold repetitive sequence of the B1 gene (5′-TCTTTAAAGCGTTCGTGGTC-3′ and 5′-GGAACTGCATCCGTTCATGAG-3′), which is found in all known parasite strains [15]. A 134-bp competitive internal standard containing the same primer template sequences as the 194-bp B1 PCR fragment was synthesized, and amplified along with parasite DNA. Amplification was performed using a 50 µl reaction mixture containing 1.24 U Amplitaq DNA polymerase, 1X PCR buffer (Promega, Madison, WI), 0.2 mM each of dGTP, dATP, dTTP, and dCTP, and 0.4 mM of each B1 primer. For each reaction, a known amount of DNA from the tissues was amplified with varying amounts of the internal standard. The levels of parasite load were estimated by comparison to the internal controls.

### Cytotoxic T lymphocyte (CTL) assay

CTL assay was performed as previously described [6]. Briefly CD8^+^ T cells stimulated TLA were incubated with infected ^51^Cr labeled macrophages at various effector-target ratio in 96 well U-bottomed plates. After 4 hr incubation the supernatants were measured for radioactive release and percentage of cytotoxic response calculated.

### Statistical analysis

Differences in percentage, absolute number, MFI and parasite burden were evaluated using Student's *t* test with P<.05 taken as statistically significant. Error bars in graphs represent standard deviation of values of individual mice in the group from one experiment. Comparison of survival curves was performed using Log-rank (Mantel-Cox) Test. For statistical analysis of CTL data, 2-way ANOVA was used. All computations were performed using GraphPad Prism Software.

## Results

### Anti IL-7 treated KO mice fail to mount a potent splenic CD8^+^ T cell response

To determine the role of IL-7 and IL-15 in the induction of primary CD8^+^ T cell response against *T. gondii* infection, anti IL-7 antibody was administered to WT and IL-15^−/−^ mice infected with *T. gondii*. Since a previous study has demonstrated significantly increased CD44 on T cells as early as 1 week post parasite challenge [16], T cell activation in these animals was evaluated by measuring the surface expression of this molecule. While naïve wild-type (WT) mice had a higher percentage of CD44^int/hi^ CD8^+^ T cells than uninfected IL-15 knock-out (KO) mice, we did not observe significant differences in CD44^int/hi^ frequency between infected WT and KO mice. Since IL-7 is considered to be important for prolonged survival of naïve CD8^+^ T cells, neutralization of this cytokine in WT mice could possibly lead to defective CD8^+^ T cell activation in these animals [17]. Interestingly, as shown in Figure. 1*A*, anti IL-7 antibody treatment of WT mice did not result in attenuation of CD8^+^ T cell response against *T. gondii*. This is consistent with similar findings reported by Ku *et al* in a non-infectious disease model where treatment of WT recipient animals with anti IL-7 in combination with anti-IL7Rα had no clear effect on adoptively transferred CD8^+^ T cells [18]. This suggests that absence of IL-15 or IL-7 alone does not affect CD8^+^ T cell activation during acute Toxoplasmosis. However, IL-7 blockade in KO mice attenuated CD44 up-regulation on CD8^+^ T cells (Figure 1*A*), implying that absence of both the cytokines affects their activation.

As previous studies including those from our laboratory have demonstrated that IFN-γ producing CD8^+^ T cells play a crucial role in maintaining long-term immunity against *T. gondii* [4], [19], we determined if presence of both IL-7 and IL-15 are needed for optimal induction of this subset in response to antigenic stimulation. As shown in Figure 1*B*, while reduced number of CD8^+^IFNγ^+^ T cells was observed in IL-15 deficient mice, IL-7 depleted WT mice did not exhibit a defect in the generation of this population. Interestingly, anti IL-7 treated KO mice exhibited a nearly 80% decrease in absolute number of CD8^+^IFNγ^+^ T cells vis-à-vis untreated controls (Figure 1*B*). However, the reduced number of CD8^+^IFNγ^+^ T cells could be a downstream consequence of lower number of activated CD8^+^ T cells. To address this possibility we measured IFN-γ expression in CD8^+^CD44^int/hi^ population in the infected animals. As shown in Figure 1*C* (top panel), majority of the IFN-γ producing CD8^+^ T cells were present in the CD44^int/hi^ subset. In agreement with our results in Figure 1*B*, IL-7 blockade in IL-15 KO mice reduced the frequency of IFN-γ expressing cells in the CD44^int/hi^ subset (Figure 1*C*). In contrast to our data in Figure 1*B*, IL-15 deficiency alone did not down-regulate the frequency of IFN-γ producing CD8^+^ T cells (Figure 1*C*). This apparent discrepancy can be explained by the fact that IL-15 is needed for the survival of both memory and naïve CD8^+^ T cells and absence of this cytokine, can affect the survival of both these subsets [20]. This is further supported by another study, in which continuous treatment of thymectomized mice with anti IL-7R antibody led to only minor reduction in naïve CD8^+^ T cells, suggesting that factors other than IL-7 can support the survival of these cells [21].

As role of IL-7 and IL-15 alone in mediating CD8^+^ T cell proliferation has been recently demonstrated in non infectious disease models [22], [23], next, we determined if reduced CD8^+^ T cell response in anti IL-7 treated KO mice infected with *T. gondii* is due to defect in their proliferative ability. To address this issue, absolute number of CD8^+^ T cells in the splenic population was measured by intracellular staining. Ki-67, a protein expressed in non-G_0_ phases of cell cycle has been shown to be essential for cell cycle progression [24]. As shown in Figure 1*D*, KO mice infected with parasite exhibited a significant decrease in the absolute number of actively cycling splenic CD8^+^ T cells that was severely exacerbated by anti IL-7 antibody treatment of these mice. To further investigate the proliferation of activated CD8^+^ T cells, we assessed the percentage of CD8^+^Ki-67^+^ T cells by gating on CD8^+^CD44^int/hi^ population. Similar to the observations made in Figure 1*C*, absence of IL-15 alone did not significantly alter the percentage of CD8^+^CD44^int/hi^ Ki-67^+^ T cells (Figure 1*E*). In agreement with observations made above, IL-7 depletion of KO mice led to substantial decrease in cycling CD8^+^CD44^int/hi^ T cells. Conversely, anti IL-7 treatment of WT mice failed to make any significant changes in this subset of CD8^+^ T cells (Figure 1*E*).

Apart from IFN-γ production, another critical effector function of CD8^+^ T cells is their ability to lyse infected target cells [25]. Cytotoxic function of immune CD8^+^ T cells generated in response to *T. gondii* infection has been well demonstrated by studies conducted by our group and other laboratories [6], [25], [26]. To determine the effect of IL-7/IL-15 on the development of CTL response against *T. gondii*, infected WT and KO animals were treated anti IL-7 antibody. As expected, purified CD8^+^ T cells from saline treated WT mice exhibited a strong CTL activity, which was unaltered by anti IL-7 treatment of these animals (Figure 1*F*). Similarly, normal CTL activity in response to *T. gondii* infection was observed in KO mice. However, administration of anti IL-7 to the KO animals severely reduced their CTL response (Figure 1*F*) further emphasizing that the absence of both IL-7 and IL-15 can cause a severe impediment in the development of antigen-specific CD8^+^ T cell immunity against Toxoplasma infection.

### Splenic CD8^+^ T cells from anti IL-7 antibody treated KO mice exhibit sub-optimal Bcl-2 up-regulation

Development of a potent CD8^+^ T cell response involves an appropriate balance between cell survival, apoptosis and proliferation [27], [28], [29], [30]. One of the most widely studied regulators of cell survival is Bcl-2, an anti-apoptotic molecule [27]. Over-expression of Bcl-2 has been shown to prevent apoptosis by blocking the release of cytochrome *c* from mitochondria [31]. Previous studies using Bcl-2^−/−^ mice have clearly demonstrated the critical role played by this molecule in peripheral T cell maintenance [32]. As expression of Bcl-2 and IL-7R has been associated with long-lived CD8^+^ T cell development, we studied expression of these two molecules on CD8^+^ T cells of *T. gondii* infected mice [33]. Activated CD8^+^CD44^int/hi^ T cell population from infected animals was assayed for Bcl-2 by intracellular staining. As shown in Figure 2*A* *,* irrespective of anti IL-7 treatment, similar levels of Bcl-2 expression on the CD8^+^ CD44^int/hi^ T cells of WT mice was observed. However, KO mice exhibited a modest reduction in the levels of this molecule, which was severely down-regulated in anti IL-7 antibody treated KO mice (Figure 2*A*).

Recent studies have demonstrated that memory precursor effector cells exhibit high levels of Bcl-2 and can be identified by IL-7Rα^hi^ (CD127) expression [33]. In the *T. gondii* model, since preferential CD127 expression has been shown to be a hallmark of memory precursor CD8^+^ T cells i.e. effector CD8^+^ T cells that are destined to become memory CD8^+^ T cells [25], lower Bcl-2 expression in anti IL-7 treated KO mice could be due to decreased frequency of CD127^hi^ cells in these animals. To address this possibility we performed a 4-color FACS analysis and examined the CD8^+^CD44^int/hi^ subset for Bcl-2 and CD127 expression. While we observed a similar percentage of CD44^int/hi^ cells expressing CD127 in WT animals treated with or without antibody, (Figure 2*B*), anti IL-7 treatment of KO animals showed a modest decrease in CD127 expressing cells in comparison to other groups. As shown in Figure 2*B*, in all the experimental groups, majority of Bcl-2 expressing cells belonged to the CD127^hi^ population. Moreover, while moderate reduction in percentage of Bcl-2^+^ cells within CD127^hi^ population of KO mice was noted, anti IL-7 treatment further down-regulated this response (Figure 2*B*). Conversely, IL-7 depletion of WT animals did not significantly alter the frequency of CD127^hi^ cells expressing Bcl-2. Since majority of Bcl-2^+^ cells co-expressed high levels of CD127, we wanted to evaluate if the patterns of Bcl-2 expression levels in CD127^hi^ subset were similar to CD44^int/hi^ cells. In agreement with observations made in Figure 2*A* and 2*B*, lack of IL-15 or IL-7 resulted in moderate or no reduction in Bcl-2 levels respectively (Figure 2*C*). However, IL-7 depletion in KO mice caused a significant decrease of Bcl-2 MFI in CD8^+^CD44^int/hi^ CD127^hi^ T cells. Next we evaluated the differences in the expression of Bcl-2 molecule within CD127^hi^Bcl-2^+^ population of WT and KO animals treated with or without antibody. As shown in Figure 2*D*, lack of either IL-15 or IL-7 did not affect Bcl-2 up-regulation in CD8^+^CD44^int/hi^CD127^hi^Bcl-2^+^ T cells, while absence of both cytokines severely down-regulated the expression of this molecule in this subset. Combined, our data suggests that in absence of both IL-7 and IL-15 development of CD8^+^CD127^hi^Bcl2^+^ T cell subset is severely compromised.

### Lack of both IL-7 and IL-15 results in suboptimal expansion of BrdU labeled splenic CD8^+^ T cells in *T. gondii* infected animals

Since Bcl-2 has been shown to be critical for peripheral CD8^+^ T cell survival, reduced effector functions of CD8^+^CD44^int/hi^ cell in anti IL-7 treated KO mice could be due to decreased survival of these cells. To address this issue, we adopted a BrdU “pulse-chase” approach that would permit enrichment of antigen-specific cells in the BrdU labeled subset. This strategy would allow us to study maturation, effector functions and survival of antigen-specific CD8^+^ T cells. Antibody treated and untreated KO mice were injected with BrdU for 4 days, then subsequently CD8^+^ T cells response in the BrdU labeled population was measured at day 6 and 14 p.i. (Figure 3*A*). As shown in Figure 3*B* and Figure 3*C*, anti IL-7 treatment of the knock out animals did not have an effect on absolute number or frequency of BrdU^+^ CD8^+^ T cells at day 6 p.i. However, at day 14 p.i., continued blockade with anti IL-7 dramatically reduced both the absolute number and frequency of these cells. Interestingly, BrdU^+^ CD8^+^ T cell response in the antibody treated group at day 14 p.i. was also significantly lower than that at day 6 p.i.. This data suggests that continued absence of both IL-7 and IL-15 leads to sub-optimal CD8^+^ T cell response in *T. gondii* infected animals.

### Blockade of IL-7 in KO mice does not severely impair maturation or IFN-γ production or cell cycling of BrdU^+^ CD8^+^ T cells

Next, we wanted to address if reduced CD8^+^ T cell response observed in bulk population (Figure 1) was also evident in the BrdU labeled cells. As shown in Figure 4*A* at day 6 p.i., 51–55% of BrdU^+^ CD8^+^ T cells in both antibody treated and untreated KO animals expressed high levels of CD44. Similarly, no difference in the expression of this activation marker in the BrdU labeled subset of anti IL-7 antibody and untreated animals was observed at day 14 p.i. (Figure 4*A*), suggesting that absence of IL-7 in KO mice does not inhibit CD8^+^ T cell maturation. As *in vitro* addition of IL-7 to human T cells has been shown to up-regulate IFN-γ expression in a dose dependent manner [34], next we determined if neutralization of IL-7 affects the IFN-γ production of BrdU labeled CD8^+^ T cells. As shown in Figure 4*B*, irrespective of IL-7 depletion, 30–34% of BrdU^+^ CD8^+^ T cells expressed IFN-γ at day 6 p.i. and this percentage increased to 61–66% at day 14 p.i.. Since a recent study in LCMV model has shown that IL-7 has a role in CD8^+^ T cell proliferation, we examined whether a reduced frequency of BrdU labeled CD8^+^ T cells in the cytokine depleted animals at day 14 p.i. could be attributed to reduced expansion of these cells [35]. To determine if this is the case, Ki-67 expression was measured by flow cytometry. As shown in Figure 4*C*, while anti IL-7 treatment had no significant effect on cell cycling at day 6 p.i., moderate reduction in this group was observed at day 14 p.i. These results suggest that attenuated proliferation as a result of IL-7 depletion in KO mice plays a minor role in severely diminished frequency of BrdU^+^ CD8^+^ T in Toxoplasma infected animals.

### Blockade of IL-7 in KO mice down-regulates Bcl-2 and IL-7Rα up-regulation of BrdU^+^ CD8^+^ T cells

To determine if lower Bcl-2 levels observed in the activated phenotype (CD44^int/hi^) CD8^+^ T cells from anti IL-7 treated KO mice (Figure 2) is due to reduced frequency of antigen-specific cells in these animals, BrdU labeled cells were assayed for Bcl-2 and CD127, hallmarks of effector cells destined to be long-lived memory cells [33]. As shown in Figure 5*A*, IL-7 depletion at day 6 p.i. did not make a significant difference in the percentage or expression of CD127 or Bcl-2 within BrdU^+^CD8^+^ T cell population. Concomitant with a decrease in the percentage of CD127^hi^CD8^+^ T cells, antibody blockade at day 14 p.i. caused a reduction in Bcl-2 MFI in BrdU^+^CD8^+^ T cells and BrdU^+^CD127^hi^CD8^+^ T cells (Figure 5*A*–*C*). Interestingly, we found that depletion of IL-7 in the KO mice led to a profound down-regulation in the development of CD127^hi^Bcl-2^hi^ cells within BrdU^+^ population (Figure 5*A*), and reduction of Bcl-2 expression within these cells was even more pronounced in the Bcl-2^hi^ subset (Figure 5*D*). Conversely, 3 fold higher percentage of CD127^hi^Bcl-2^hi^ population was observed in untreated control animals (Figure 5*A*) and increase of Bcl-2 levels within these cells from these animals over time correlated with an increase in CD127 expression (MFI of 218–232 to 356–367) (Figure 5*A*). In contrast, anti IL-7 treatment of KO mice led to minimal increase in CD127 MFI in the above subset and expression of this molecule merely rose from a MFI of 210–236 at day 6 p.i. to a MFI of 251–260 at the later time point (Figure 5*A*). These observations demonstrate that while lack of IL-7 or IL-15 alone has little impact on IL-7Rα and Bcl-2 up-regulation within CD8^+^ T cell population during acute Toxoplasmosis, absence of both IL-7 and IL-15 leads to defective expression of these molecules.

### In absence of IL-7, KO mice fail to control *T. gondii* infection

As mentioned earlier the role of CD8^+^ T cells in immuno-protection against *T. gondii* infection is well documented [1], [3], [19]. Since depletion of IL-7 in KO mice generated poor CD8^+^ T cell response against *T. gondii* infection, next we determined, if deficiency of both these cytokines reduced the host survival against the parasite. One day prior to infection WT and KO animals were administered anti IL-7 antibody or equal volume of saline via i.p. route. The treatment was subsequently continued at 3-day intervals until the termination of experiment. As shown in Figure 6*A*, while 20% and 50% mortality was noted in control KO mice and anti IL-7 treated WT mice respectively, all the animals treated with anti IL-7 antibody succumbed to infection by day 26 p.i..

To confirm that high mortality observed in anti IL-7 treated KO mice is due to their compromised capacity to clear parasites, tissues (gut, spleen, liver and lung) from these animals were analyzed for parasite load by quantitative PCR at day 14 p.i. [36]. As shown in Figure 6*B*, anti IL-7 treatment of KO mice severely reduced their ability to clear infection as maximal parasite burden was observed in the tissues of these animals. This data suggests absence of both IL-7 and IL-15 severely impairs host resistance to *T. gondii* by diminishing parasite clearance.

### In absence of IL-7, IL-15^−/−^ mice infected with replication incompetent *T. gondii* parasites show attenuated CD8^+^ T cell response

A recent study using two photon laser microscopy has demonstrated that memory T cells can be infected by *T. gondii* by direct transfer of parasites from neighboring cells [37]. Considering the high parasite burden observed in IL-7 depleted KO mice, poor CD8^+^ T cell response in these animals might be a direct consequence of parasitized CD8^+^ T cells. In order to circumvent this issue, we used cps1-1 parasites, a replication incompetent strain of *T. gondii*, which are deficient for *de novo* pyrimidine biosynthesis [38]. Using a similar IL-7 blocking regimen, KO mice were infected with 1×10^6^ cps1-1 parasites via i.p. route. At day 14 p.i. both antibody treated and untreated controls were sacrificed and spleens assayed for CD8^+^ T cell response. As shown in Figure 7*A*, IL-7 depletion of naïve mice did not produce any change in absolute number of CD8^+^CD44^int/hi^ T cells. Similar observations were made even with 3 fold higher doses of the blocking antibody, suggesting that unaffected CD8^+^ T cell development in anti IL-7 treated naïve mice was not due to incomplete IL-7 depletion (data not shown). However, similar to observations made with replication competent parasites, antibody treatment of cps1-1 infected KO mice severely attenuated the CD8^+^CD44^int/hi^ T cell response in these animals (Figure 7*A*). Moreover IFN-γ production by CD8^+^ T cells upon both anti CD3 (Figure 7*B*) or TLA (data not shown) stimulation in cps1-1 infected mice was also diminished as a result of anti IL-7 treatment. However even with polyclonal anti CD3 stimulation no significant difference in percentage of IFN-γ producing CD8^+^ T cells between naive KO mice treated with or without antibody was observed (Figure 7*B*). Overall, the extent of attenuation of CD8^+^ IFN-γ^+^ T cell response due to IL-7 depletion exhibited similar trends in both cps1-1 and ME49 infected KO mice, suggesting that high parasite burden in ME49 infected antibody treated KO mouse does not significantly contribute to the decreased CD8^+^ T cell response in these animals. Significantly, considering the utility of cps1-1 as a live attenuated vaccine, it makes our current findings particularly relevant for improving vaccination regimens against Toxoplasma.

## Discussion

The results presented in the current studies demonstrate that both IL-7 and IL-15 play an important role in the induction of primary CD8^+^ T cell response against *T. gondii* infection. An earlier report from our laboratory has pointed towards the importance of exogenous IL-7 treatment in the generation of primary CTL vivo, however the extent of involvement of endogenous levels of this cytokine was not described [6]. Although, the role of related γ_c_ cytokine IL-15, in the generation and maintenance of long-term CD8^+^ T cell immunity against *T. gondii* is well established by several studies conducted in our laboratory, importance of this cytokine in the initiation of primary CD8^+^ T cell response has not been studied [7], [11], [12]. In the present study we demonstrate that while lack of IL-15 or IL-7 alone has minimal or no impact on CD8^+^ T cell development, the absence of both cytokines severely impairs this response.

In the present study we demonstrate that depletion of IL-7 alone did not impede the induction of primary CD8^+^ T cell response in the WT animals. Similarly, lack of IL-15 did not cause significant deficits in CD8^+^ T cell maturation, IFN-γ production, proliferation and cytolytic function of infected animals. However, the major defect caused by lack of IL-15 lay in the attenuated number of CD8^+^ T cells generated in KO mice in response Toxoplasma challenge. In agreement with observations by Schluns *et al* in VSV model, our findings strongly suggest that IL-15 is essential for regulating CD8^+^ T cell burst size during acute Toxoplasmosis, while its role in acquisition of effector functions is minimal [9]. Interestingly, depletion of IL-7 in KO mice had a profound effect on the generation of a potent CD8^+^ T cell response in these animals. This is manifested by their attenuated maturation and inability of these cells to produce optimal IFN-γ or proliferate or lyse parasite infected target cells. Based on these observations it appears that IL-7 and IL-15 together, are the key mediators of maturation and effector function development of CD8^+^ T cells during acute *T. gondii* infection.

Apart from functionality and maturation, Bcl-2 mediated CD8^+^ T cell survival is one of the critical determinants of optimal response against a pathogen. Several studies have established the important roles played by IL-7 and IL-15 respectively in mediating Bcl-2 up-regulation in lymphocytes [39], [40]. However the effect of both these cytokines on Bcl-2 and IL-7Rα expression in CD8^+^ T cells during acute phase of infection remains understudied. In the present study we demonstrate that IL-7 depletion alone had no effect on Bcl-2 up-regulation which is consistent with earlier studies conducted with Vesicular Stomatitis Virus (VSV) model of infection [41]. Further, our data suggests that absence of IL-15 alone may play a minor role in committing CD8^+^CD44^int/hi^CD127^hi^ T cells to express Bcl-2 during the acute phase of infection. This is the first report that demonstrates in an infectious disease model that lack of both IL-7 and IL-15 not only down-regulates the development of CD127^hi^ subset, but also reduces the frequency of CD127^hi^ cells expressing Bcl-2. Moreover, it has been recently shown that IL-15 down-regulates pro-apoptotic Bax, an intermediate in TRAIL mediated apoptosis pathway, and increases anti-apoptotic molecules in CD8^+^ T cells [42], [43]. Hence it is possible that in absence of both IL-7 and IL-15, lower Bcl-2 levels may be secondary to increased TRAIL mediated signaling in these animals.

A caveat of conclusions gleaned from analysis of activated phenotype CD8^+^ T cells is that in anti IL-7 treated KO mice only 21–24% of these cells produce IFN-γ in an antigen-specific manner. Hence observations made regarding Bcl-2 or CD127 expression in activated phenotype CD8^+^ T cells in the above animals may not be as relevant to *T. gondii* specific response. Moreover, the observed differences in activated CD8^+^ T cell response as a result of IL-7 depletion in KO mice could be the result of decreased antigen-specific CD8^+^ T cells due to potentially reduced naïve CD8^+^ T cell survival in the these animals. As stated earlier, importance of IL-7 in naïve T cells survival has been documented [44]. Although we did not observe significant difference in absolute number or frequency of CD8^+^ T cells (data not shown) or CD8^+^CD44^int/hi^ T cells as a result of anti IL-7 treatment in uninfected KO mice after 2 weeks of antibody treatment, the possibility of altered survival of naïve T cells in infected mice cannot be entirely ruled out. To circumvent this issue, we utilized a BrdU pulse-chase approach that would enable us to track endogenous CD8^+^ T cells highly enriched for *T. gondii* specific cells. Although it is possible for non-specific HP (homeostatic proliferation) memory CD8^+^ T cells to incorporate BrdU, a recent study has shown that true memory or antigen experienced memory phenotype CD8^+^ T cells are 8 times more efficient at expanding than HP memory T cells [45]. Our results demonstrate that IL-7 depletion in KO mice does not affect CD8^+^ T cell priming as evident by similar BrdU labeled CD8^+^ T cell response at day 6 p.i. However, lack of this cytokine prevents optimal survival of these cells at a later time point. Interestingly, absence of both IL-7 and IL-15 does not adversely affect CD44 expression, IFN-γ production or cell cycling at either day 6 or day 14 p.i. suggesting that reduced effector function and maturation are primarily consequences of poor survival of antigen-specific CD8^+^ T cells in these mice. This was suggested by the observation that anti IL-7 treatment of KO mice led to decreased expression of Bcl-2 and CD127 in the BrdU^+^ population. However, considering the high pathogen burden in these mice, it needs to be investigated in future studies whether one of the factors contributing to poor development of memory CD8^+^ T cells in IL-7 depleted KO mice is accelerated exhaustion of CD8^+^ T cells via PD-1-PDL-1 pathway [46], [47].

Apart from CD8^+^ T cells, Natural Killer (NK) cells and CD4^+^ T cell produce IFN-γ, a critical mediator of resistance against *T. gondii* [48]. However in *T. gondii* infection, NK cells have been shown to have a minimal protective role even during the acute phase of infection [49]. Paradoxically, despite normal splenic CD8^+^ T cell response, anti IL-7 treated WT mice exhibited elevated parasite load and mortality. This may be owing to depressed CD8^+^ T (Figure S1) cell and NK (Figure S2) cell response noted in liver in both anti IL-7 treated WT and KO (more severe defects) mice. Based on a recent report from our laboratory demonstrating that NK-dendritic cell cross-talk is critical for optimal CD8^+^ T cell development [50], on-going studies in our lab are addressing whether defective hepatic CD8^+^ T cell response is secondary to sub-optimal NK cell development and why hepatic but not splenic NK cell response is IL-15 dependent.

Although in several infectious disease models including *T. gondii*, primary CD8^+^ T cell response during acute infection phase has been shown to be independent of CD4^+^ T cell help, the role of CD4^+^ T cells themselves in partially mediating protection against *T. gondii* cannot be discounted [51], [52]. Nevertheless, we did not observe any difference in CD4^+^ T cell response between untreated and anti IL-7 injected KO mice at day 14 p.i. (data not shown). Thus, the above data strongly suggests that poor survival and high parasite burden in antibody treated KO mice are primarily consequences of sub-optimal CD8^+^ T cell development in these mice.

Since a previous study has demonstrated that IL-2 down-regulates IL-7Rα expression on activated T cells, it is possible that in the absence of IL-7 and IL-15, other cytokines may be involved in down regulating CD8^+^ T cell response [53]. Unlike other pathogens, *T. gondii* infection does not induce a potent IL-2 response even during the acute phase of infection [6], [54], [55], [56]. Hence, it is not surprising that no significant difference in IL-2 producing cells was noted between untreated and antibody treated KO mice (Figure S3). In addition to IL-7, IL-15 and IL-2, IL-12 is another important cytokine known to regulate the development of memory CD8^+^ T cell response. A recent study by Pearce *et al* has demonstrated that high IL-12 levels induced during *Listeria monocytogenes* infection down-regulates CD8^+^ memory T cell differentiation [57]. However, in the present study administration of anti IL-7 to KO mice did not alter their IL-12 production or the expression IL-12Rβ1 and IL-12Rβ2 by CD8^+^ T cells (Figure S4). Apart from IL-7 and IL-15, potential role of other γ_c_ family members like IL-4 [58], [59] and IL-21 [60] that have been implicated in CD8 memory generation in other infectious disease models, need to be considered in future investigations of memory CD8^+^ T cell development during toxoplasmosis.

Recent study by Klonowski *et al* with VSV using IL-7 deficient animals has shown that CD127 and Bcl-2 up-regulation are IL-7 independent [41]. However our observations demonstrate that in absence of IL-15, endogenous IL-7 has a non-redundant role in mediating Bcl-2 and CD127 up-regulation. Alternatively, it is possible that in absence of both IL-7 and IL-15, higher dependence on related γ_c_ family member, IL-21 may decrease CD127 and Bcl-2 expression on CD8^+^ T cells [61]. Taken together, our data suggests that IL-7 and IL-15 have overlapping roles in mediating up-regulation of Bcl-2 and CD127 on antigen-specific CD8^+^ T cells and that differential CD127 expression does not impede CD44 expression or effector functions during acute phase infection.

The findings presented in the current manuscript go a long way in understanding synergistic effect of IL-7 and IL-15 in the development of CD8^+^ T cell immunity against *T. gondii* infection. However, the molecular mechanism regulating this synergism remains to be further investigated. Preliminary studies from our laboratory suggest that in absence of both of these cytokines there is defective up-regulation of master transcriptional factors, T-bet and Eomes in CD8^+^ T cells (Figure S5). This defect in T-bet up-regulation is evident as early as day 6 p.i. (Figure S5). Significance of this down-regulated T-bet and Eomes expression will have to await further investigation. Nevertheless, the data presented in this manuscript provide significant insights into the combined role of IL-7 and IL-15 in the development of CD8^+^ T cell immunity during acute Toxoplasma infection. Moreover, these findings have important implications in understanding the use of IL-7 and IL-15 as adjuvant for therapeutic vaccination regimen against intracellular pathogens where CD8^+^ T cells constitute a critical component of protective immunity.

## Supporting Information

Figure S1 Anti IL-7 treated WT or KO mice exhibit attenuated hepatic CD8^+^ T cell response. A, Absolute number of CD8^+^ T cells in liver was evaluated in antibody or saline treated WT and KO mice at day 14 pi. The data is representative of one of 2 experiments with at least 3–4 mice per group.
(0.61 MB EPS)

Figure S2 Anti IL-7 treatment WT or KO mice results in reduced hapatic NK cell response. A,B Asbolute number of NK cells in spleen (A) and liver (B) was evaluated in antibody or saline treated WT and KO mice. The data is representative of one of 2 experiments with at least 3–4 mice per group.
(0.73 MB EPS)

Figure S3 Simultaneous deficiency of IL-7 and IL-15 does not result in differential IL-2 production. A, Splenocytes from antibody or saline treated KO mice were assessed for IL-2 production after overnight TLA stimulation. The data is representative of one of 2 experiments with at least 3–4 mice per group.
(0.65 MB EPS)

Figure S4 Simultaneous deficiency of IL-7 and IL-15 does not result in differential IL-12 production. A, Splenocytes from antibody or saline treated WT and KO mice were assessed for IL-12 production. B,C IL-12Rβ1 (B) and IL-12Rβ2 (C) expression was evaluated on splenic CD8^+^ T cells by flow cytometry. The data is representative of one of 2 experiments with at least 3–4 mice per group.
(4.36 MB EPS)

Figure S5 T-bet and Eomes expression are downregulated in BrdU^+^CD8^+^ T cells from IL-7 depleted KO mice. Splenocytes from BrdU injected anti IL-7 or saline treated KO mice were harvested at day 6 and day 14 p.i. A,B T-bet (A) and Eomes (B) expression and frequency was evaluated in both BrdU^+^ and BrdU^−^ CD8^+^ T cells by intracellular staining. Numbers within brackets represent MFI. The data is representative of one of 2 experiments with at least 3–4 mice per group.
(1.11 MB EPS)
